# Improved microarray gene expression profiling of virus-infected cells after removal of viral RNA

**DOI:** 10.1186/1471-2164-9-221

**Published:** 2008-05-14

**Authors:** Matthijs Raaben, Penn Whitley, Diane Bouwmeester, Robert A Setterquist, Peter JM Rottier, Cornelis AM de Haan

**Affiliations:** 1 Virology Division, Department of Infectious Diseases and Immunology, Faculty of Veterinary Medicine, Utrecht University, Yalelaan 1, 3584 CL Utrecht, The Netherlands; 2 Ambion Inc, Research & Development, 2170 Woodward St, 78744 Austin, TX, USA; 3 University Medical Center Utrecht, PO Box 85060, 3508 AB Utrecht, The Netherlands

## Abstract

**Background:**

Sensitivity and accuracy are key points when using microarrays to detect alterations in gene expression under different conditions. Critical to the acquisition of reliable results is the preparation of the RNA. In the field of virology, when analyzing the host cell's reaction to infection, the often high representation of viral RNA (vRNA) within total RNA preparations from infected cells is likely to interfere with microarray analysis. Yet, this effect has not been investigated despite the many reports that describe gene expression profiling of virus-infected cells using microarrays.

**Results:**

In this study we used coronaviruses as a model to show that vRNA indeed interferes with microarray analysis, decreasing both sensitivity and accuracy. We also demonstrate that the removal of vRNA from total RNA samples, by means of virus-specific oligonucleotide capturing, significantly reduced the number of false-positive hits and increased the sensitivity of the method as tested on different array platforms.

**Conclusion:**

We therefore recommend the specific removal of vRNA, or of any other abundant 'contaminating' RNAs, from total RNA samples to improve the quality and reliability of microarray analyses.

## Background

In various research fields, microarray analysis is frequently being used as a tool to analyze alterations of the transcriptome in response to different stimuli. However, this technology often has serious limitations related to its sensitivity, specificity and reproducibility [1]. A key step in the generation of reliable data involves the isolation and processing of the RNA, since high quality RNA is needed to obtain accurate results.

In the field of virology, microarrays are often used as a diagnostic tool to detect the presence of certain viruses within biological samples or to discover new viruses [2,3]. In addition, microarray analysis is regularly applied to identify host genes of which the expression is altered upon virus infection. Whole-genome profiling of virus-infected cells, in combination with other large-scale, high-throughput technology, rapidly increases our knowledge of virus-host interactions and may eventually lead to the production of new antivirals [4]. Often these screens are performed using cultured cells with high doses of virus in order to infect all the cells present. As most viruses replicate and transcribe their genome very efficiently, the resulting high levels of viral RNA (vRNA) may be expected to interfere with the microarray analysis. The potential interference of vRNA with array procedures is of particular concern when the vRNAs are polyadenylated, given that most microarray protocols involve mRNA amplification using oligo(dT) primers, thereby hence also amplifying viral mRNAs. This potential problem especially holds true for RNA viruses, including coronaviruses (CoV), the replication of which has been shown to result in an exponential increase in vRNA levels within hours after inoculation of the cells [5-7].

**Figure 1:**
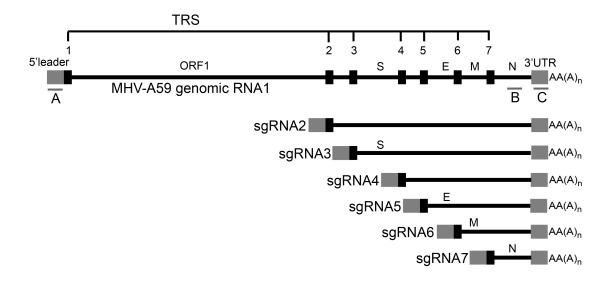
**Coronavirus RNA replication strategy**. MHV-A59 produces a nested set of subgenomic mRNAs (sgRNAs) that all have identical 5' and 3' terminal ends. Via discontinuous transcription, during the synthesis of the minus strand, sgRNAs obtain the 5' leader sequence fused to a transcriptional regulating sequence (TRS, indicated by the numbered small black boxes), followed by a protein-coding region that is flanked at the 3' terminus by an untranslated region (3'UTR) and a polyA-tail (indicated as AA(A)_n_). Note that the nucleocapsid (N) gene is present in all sgRNAs. The three biotinylated oligonucleotides, which are used in the vRNA removal procedure and which are complementary to three regions in the viral genome, are indicated by the underscores and the letters A, B, and C (see Table 1 for the nucleotide sequences).

CoVs are enveloped, positive-stranded RNA viruses and are well-known pathogens in man and animals. Their relevance has increased significantly with the current surfacing of new human CoVs (HCoVs), such as the severe acute respiratory syndrome (SARS)-CoV [8], HCoV-NL63 [9], and HCoV-HKU1 [10]. CoVs replicate exclusively within the cytoplasm of their target cells, producing a nested set of subgenomic mRNAs (Fig. 1), which contain identical 5' and 3' terminal sequences [11]. These mRNAs are transcribed by a discontinuous transcription mechanism; they acquire a 5' cap structure and become 3' polyadenylated, which makes them equivalent to host cellular mRNAs [12]. Gene expression profiling of coronavirus-infected cells has been performed in several microrarray studies in order to get more insight in the coronavirus-host interactions that contribute to pathogenesis [7,13-15].

Although large numbers of genes with altered expression have been identified in virus-infected cells, no report exists, which addresses to what extent the high levels of vRNA affect microarray performance. Therefore we investigated the potentially disturbing effect of vRNA overrepresentation on array outcome by using the mouse hepatitis coronavirus (MHV) as a model and by employing two different microarray platforms. Our observations indeed show that the presence of vRNAs in the preparation interferes quite dramatically with the genechip assays. Removal of vRNAs from the total RNA pool before the processing of the RNA for subsequent array hybridization drastically decreased the number of false positive hits for one platform and increased overall sensitivity in both systems. We conclude that depleting vRNAs from infected cell total RNA extracts is beneficial for the microarray analysis not only of cornavirus infection but probably for many other virus infections as well. In addition, the approach is likely to be equally advantageous in other circumstances where 'contaminating' (viral) RNAs constitute a significant fraction of the target RNA to be processed for microrarray analysis.

## Results

### vRNA interferes with microarray analysis

**Figure 2:**
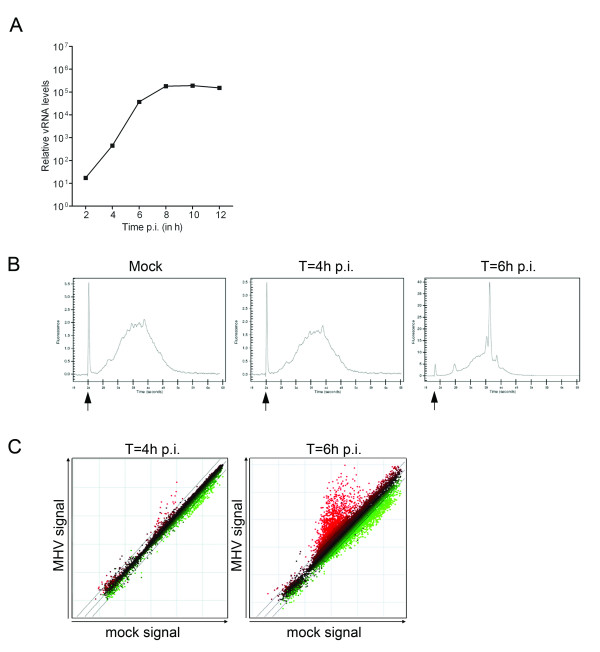
**Microarray analysis of MHV-infected LR7 cells**. (A) Genomic viral RNA (vRNA) levels in MHV-infected LR7 cells (MOI 10) were measured by quantitative RT-PCR at the indicated time points. The data are presented as relative vRNA levels. (B) Successful amplification of the mRNA within the individual samples was monitored by analyzing the cRNA samples with a Bioanalyzer (Agilent), according to the manufacturer's instructions. Representative mRNA amplification plots of total RNA samples obtained from mock- or MHV-infected cells at 4 h and 6 h p.i. are shown. The indicated plots represent the size distribution of the total mRNA content present in the samples. The marker peak is indicated by the arrow. Note that the scaling is different between the plots in order to visualize the complete profile. (C) Total RNA was isolated and processed for microarray analysis as described in the Methods section. The scatter plots display the average expression values from independent dye-swap hybridizations (n = 6) for each gene present on the arrays at the indicated time-points p.i. Red spots represent upregulated gene transcripts while green spots represent downregulated gene transcripts upon infection of cells with MHV. The dashed lines indicate the 2-fold change cut-off.

In order to investigate the potential interference of vRNA during microarray procedures, we first analyzed in detail the synthesis of vRNA during the course of an MHV infection and the subsequent effect of the presence of vRNA on the mRNA amplification plots. To this end, we infected LR7 cells with MHV-A59 (MOI = 10) and determined the amount of vRNA at different time-points p.i. using quantitative Taqman RT-PCR. As is shown in Fig. 2A, early in infection there is an exponential increase of the vRNA levels. The 4 h and 6 h p.i. time-points, which differ approximately 100 fold in their amounts of vRNA, were chosen for further analysis. Total RNA extracts obtained from mock- and MHV-infected cells were subjected to the mRNA amplification protocol, after which the resulting cRNA was analyzed with the Bioanalyzer. As previously mentioned, coronaviral RNAs are polyadenylated, and should therefore also be amplified with the oligo(dT) primers. Whereas amplification of mRNA of mock- and MHV-infected cells at 4 h p.i. resulted in similar Bioanalyzer profiles, aberrant peaks were observed after amplification of the mRNA derived from MHV-infected cells at 6 h p.i. (Fig. 2B). The most abundant peak within the latter amplification plot corresponded in size with the most abundant subgenomic RNA of MHV [16], which encodes the N protein (Fig. 1). Determination of the peak surface area showed that at least 40% of the amplified mRNA pool is of viral origin.

Next, microarray experiments were performed to study the effect of these huge amounts of vRNA present in the target cRNA samples. First, 70-mer oligonucleotide arrays, which are routinely used in the Utrecht microarray facility, were used as proof of principle. As shown in the scatter plots in Fig. 2C, expression of only few genes was upregulated at 4 h p.i., whereas there appeared to be a dramatic increase in the number of genes, the expression of which was upregulated at 6 h p.i. However, quantitative RT-PCR could not confirm the differential expression of several of these genes (see Additional file 1; False-positive hits). For example, the very high level of expression of the Usp2 gene in MHV-infected cells at 6 h p.i., according to the array analysis, could not be validated by quantitative RT-PCR. In addition, expression of several other genes, which were identified as highly upregulated by the array analysis, could not be detected by Taqman RT-PCR both in infected and mock-infected cells. Interestingly, when the nucleotide sequences of the oligonucleotides on the arrays, of these apparently false-positive hits, where compared to the N gene nucleotide sequence, small stretches (10–15 nucleotides) of identical sequences were observed (data not shown), substantiating the suggestion of cross-hybridization of vRNA to specific sequences on the arrays.

### Cross-hybridization of vRNA to arrays

**Figure 3:**
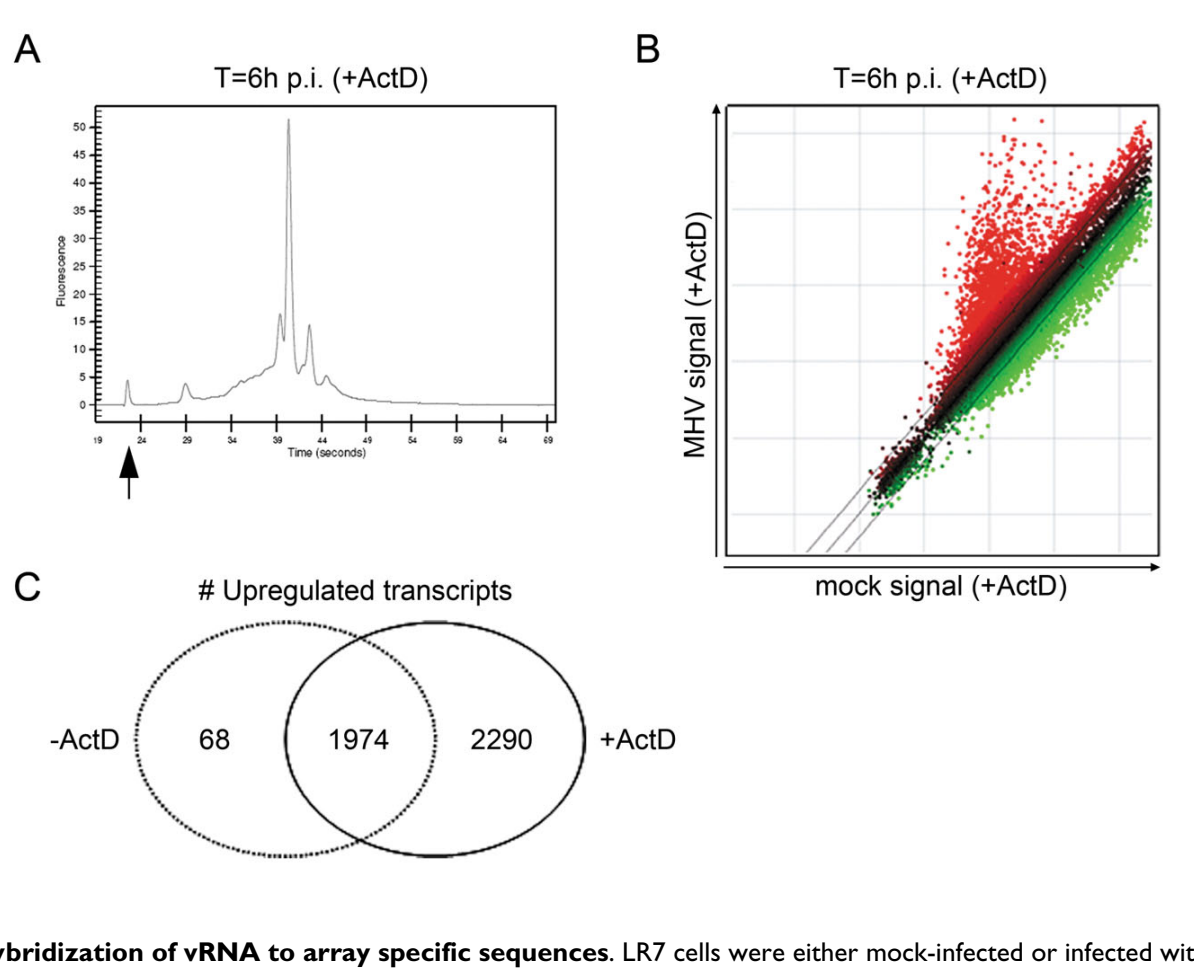
**Cross-hybridization of vRNA to array specific sequences**. LR7 cells were either mock-infected or infected with MHV (MOI 10). The cells were incubated with ActD (20 μg/ml) 1 h prior to infection, and maintained in the presence of this drug throughout the experiment. Total RNA was isolated from mock- or MHV-infected cells at 6 h p.i. (A) A representative mRNA amplification plot of a total RNA sample derived from MHV-infected, ActD-treated cells at 6 h p.i. The arrow indicates the marker peak. (B) The scatter plot displays the average expression values from independent dye-swap hybridizations (n = 6) for each gene present on the arrays as described in legend of Fig.2. (C) The Venn diagram shows a comparison between the experiments in the absence or presence of ActD.

The cross-hybridization of vRNAs to the arrays was further explored by performing a similar experiment as described above. Only now, the mock- and MHV-infected cells were treated with ActD, which blocks cellular transcription but not MHV replication [17,18]. By using this inhibitor of cellular transcription we were able to discriminate between genuine upregulation of gene expression and cross-hybridization. Apparent upregulation of gene expression in the presence of ActD, is most likely caused by cross-hybridization of vRNAs to the arrays. Amplification of the mRNA obtained from the MHV-infected, ActD-treated cells again resulted in the appearance of aberrant peaks, which were not observed in the amplification plots of the mock-infected cells (Fig. 3A). The subsequent microarray experiment (Fig. 3B) gave similar results to the one shown in Fig. 2C. Almost 2000 genes were upregulated under both conditions, indicating that most genes identified by the array analysis are indeed the result of cross-hybridization and not of induction of gene expression (Fig. 3C). Moreover, 2290 genes were specifically identified in the experiment with ActD. Since viral mRNA is efficiently replicated and transcribed in the presence of ActD, while cellular transcription is blocked, the identification of these genes probably results from a relative increase in the amount of vRNA within the target cRNA samples. Indeed, comparison of the Bioanalyzer profiles shown in Fig. 3A and Fig. 2B showed that the aberrant peak is higher after treatment with ActD. Interestingly, there is also a significant number of genes downregulated in the absence and presence of ActD, indicating that a post-transcriptional mRNA decay pathway is induced during MHV infection, a phenomenon which we described recently [7].

### Extraction of vRNA from total RNA improves microarray analysis

**Figure 4:**
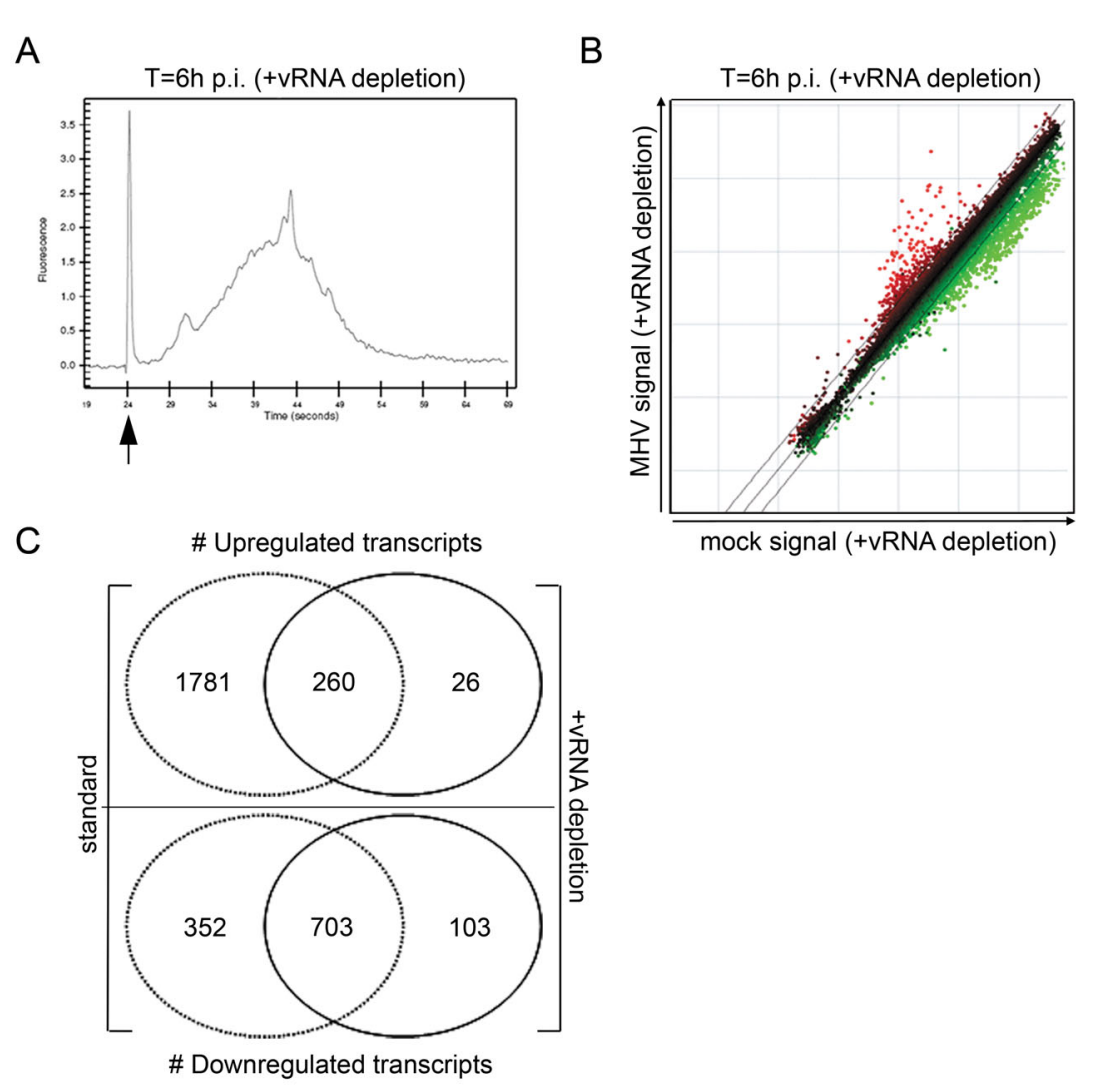
**vRNA depletion improves microarray analysis**. Total RNA samples were obtained from mock- and MHV-infected LR7 cells at 6 h p.i. were subjected to the vRNA depletion protocol as detailed in the Materials and methods section. (A) Amplification of the mRNA was monitored by analyzing the cRNA samples with a Bioanalyzer. A representative mRNA amplification plot of a total RNA sample derived from MHV-infected cells at 6 h p.i. after vRNA removal is shown. The arrow indicates the marker peak. (B) Total RNA samples were treated with the biotinylated oligo's (indicated as vRNA depleted) and were processed for microarray analysis as described in the Methods section. The scatter plots display the average expression values from independent dye-swap hybridizations (n = 6) for each gene present on the arrays as described in legend of Fig.2. (C) The Venn diagrams show a comparison between the experiments with and without vRNA depletion.

Since the vRNAs appear to affect the array analysis significantly, we employed a technology to remove these RNAs from the total RNA pool before processing of the samples for microarray analysis (i.e. mRNA amplification, labeling, and hybridization). For this purpose, biotinylated oligonucleotides (27–30 nt in length) complementary to three regions of the MHV-A59 genomic and subgenomic RNAs (the 5' leader, N, and 3' UTR region; Fig. 1) were designed (see Methods for nucleotide sequences). The same RNA samples as described above were now subjected to the vRNA removal protocol. In short, the RNA samples were incubated with the MHV-specific oligo's under stringent hybridization conditions, after which the captured vRNAs were bound to streptavidin-coated beads, and removed. Quantitative RT-PCR targeting the viral N gene, which is present in all MHV-encoded RNAs, demonstrated that approximately 90% of the vRNAs were removed by this procedure (data not shown). Amplification of mRNAs from the samples treated with the MHV-specific oligo's confirmed the removal of the vRNAs from the total RNA pool as judged from the almost complete absence of the aberrant peaks in the Bioanalyzer profiles (Fig. 4A; compare to Fig. 2B). Importantly, the vRNA capture procedure did not appear to affect the Bioanalzyer mRNA amplification plots of the mock-infected cells (data not shown). Next, the effect of the vRNA-depletion on the microarray performance was studied. Thus, microarray analysis was performed as described above except that the target samples, derived from both infected and mock-infected cells, were treated with the MHV-specific biotinylated oligo's. The results are shown in Fig. 4B. As is evident from the scatter plot, the number of apparently upregulated transcripts was greatly reduced. Increased expression of almost 1800 genes was no longer detected after vRNA depletion when compared to the standard approach (Fig. 4C, and compare scatter plots shown in Fig. 4B and Fig. 2B). This result is consistent with the notion that vRNAs can hybridize to specific sequences on the arrays. Thus, removal of vRNAs results in a significant reduction of the number of false positive hits. 260 genes were identified under both conditions. This set of genes is likely to contain several hits that are still the result of cross-hybridization, since vRNA depletion was not 100%. For example, Usp2 and Figla, two highly upregulated genes, the differential expression of which could not be confirmed by quantitative RT-PCR, were still present within this collection of genes, although the transcriptional upregulation of these genes as judged from the microarray experiment was much less pronounced (see Additional file 1; False-positive hits). 40 genes out of these 260 hits were not detected in the experiment with ActD, indicating that these genes are specifically upregulated at the transcriptional level upon infection of cells with MHV. Some of these genes were validated by quantitative RT-PCR (see Additional file 1) and have already been described elsewhere [7]. Interestingly, transcriptional upregulation of 26 genes could only be detected with the microarray experiment after removal of the vRNA. For some of these genes, we confirmed their differential expression by quantitative RT-PCR (see Additional file 1; Additional hits).

We also compared the number of downregulated transcripts in absence or presence of the vRNA capture procedure. The majority of identified genes overlapped (n = 703) between both procedures, and downregulation of several transcripts was confirmed by quantitative RT-PCR [7]. However, more downregulated transcripts were identified with the standard method (in the presence of vRNA) than with the vRNA depletion approach, 1055 versus 806 genes, respectively (Fig. 4C). Downregulation of 352 transcripts was specifically detected with standard method, while 103 genes were exclusively detected with the new approach. The reduced number of downregulated transcripts after removal of the vRNAs is likely caused by the normalization procedure, which relies on the assumption that the bulk of genes are not differentially expressed between samples [19]. Thus, a large number of false-positive hits, caused by cross-hybridization of vRNAs, will result in an overrepresentation of downregulated transcripts.

Although we cannot completely exclude that the oligo capture procedure, besides vRNAs, also extracts some cellular mRNAs, it is obvious that this method improves the accuracy and sensitivity of the array analysis significantly. First, the number of false positive hits reduced dramatically, making follow-up analyses much easier to perform. Secondly, the improved microarray approach allowed the additional detection of several differentially expressed genes, which are potentially important targets for further research.

### vRNA extraction improves Affymetrix microarray performance

**Figure 5:**
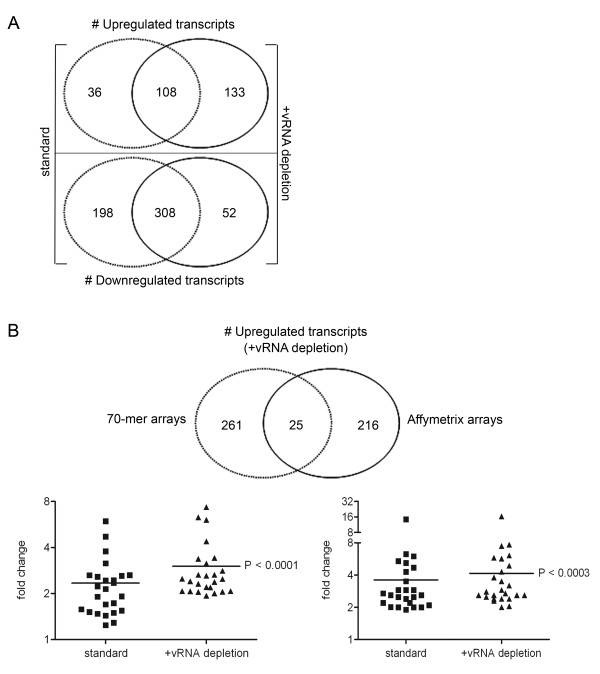
**vRNA depletion increases sensitivity on different microarray platforms**. (A) Affymetrix arrays were hybridized independently with the same target samples (t = 6 h p.i.; treated with or without vRNA depletion) as described for the 70-mer oligonucleotide arrays. Data analysis was performed as described in the Methods section. The Venn diagram depicts the comparison of the number of upregulated and, separately, the downregulated genes in the two experimental settings (with and without (standard) vRNA depletion). (B) The Venn diagram shows a comparison of the upregulated transcripts obtained with both the 70-mer arrays and the Affymetrix arrays using the vRNA depletion method. The genes within the intersection (n = 25) were selected and the fold changes in gene expression, as discovered by each array analysis, for both methods (standard versus vRNA depleted) are plotted in the graph. Note that the induced expression of several of these genes was validated by quantitative RT-PCR (see Additional file 1). A paired t-test was performed to show the significant difference between both methods for each platform.

Next we analyzed whether the removal of vRNA also improves the performance using another array platform. To this end, we used the commonly used Affymetrix GeneChips^® ^Mouse Genome 430 2.0 arrays. The same RNA samples, previously used for the experiments with the 70-mer oligonucleotide arrays, were now processed for analysis on the Affymetrix arrays as described in the Methods section. Using GeneChips^®^, the standard method detected the upregulation of 144 transcripts upon MHV infection, whereas the vRNA capture procedure resulted in the identification of 241 upregulated transcripts, 133 of which were only detected after vRNA depletion (Fig. 5A). The differential expression of some of these genes, which could only be detected after vRNA removal, was validated by quantitative RT-PCR (see Additional file 1; Additional hits). Both with the standard as with the vRNA depletion method, 308 downregulated transcripts were identified. 198 genes were excluded from detection by vRNA depletion, whereas 52 genes were specifically downregulated by using this approach. Although the problem of cross-hybridization (i.e. false-positive hits) that was observed with the 70-mer oligonucleotide arrays did not become apparent with the Affymetrix arrays, we could clearly observe an improved microarray performance with the Affymetrix arrays. Not only were more genes identified, the expression of which was induced upon infection, also the fold changes were found to be increased after vRNA clearance. This phenomenon was also observed in the experiments with the 70-mer oligonucleotide arrays. Thus, when the expression levels of the genes, identified by both array platforms using the vRNA depletion method (n = 25), were compared to the expression levels in the experiments without vRNA depletion, a significant increase in the fold changes was observed on both array platforms (Fig. 5B). The differential expression of some of these genes was validated by using quantitative RT-PCR (see Additional file 1; Hits by both platforms and both methods). Note that this observation also holds true for most other upregulated transcripts that were detected with only one array platform (data not shown). Conclusively, these results show that the removal of abundant 'contaminating' vRNAs in our CoV system enhanced the ability to detect differential expression of host mRNA using microarray technology.

## Discussion

Since microarray technology at the present has progressed towards a point where technical variation, background noise, and a lack of accuracy have greatly improved, the critical step has moved towards the preparation of the RNA sample. Non-specific hybridization is one of the problems that can frequently occur, especially when there is partial degradation of the RNA [20]. In addition, high amounts of 'contaminating' RNA are likely to add to this problem and to interfere with microarray analysis. Especially in RNA samples derived from virus-infected cells where high amounts of a limited number of 'contaminating' RNAs are to be expected. In the case of coronaviruses, replication and transcription results in the formation of a nested set of subgenomic mRNAs that are 5' capped and 3' polyadenylated (Fig. 1). In time, the amount of coronavirus vRNA increases exponentially. Most microarray protocols use mRNA amplification, which also results in amplification of coronavirus RNAs. Indeed, at 6 h post infection, substantial aberrant peaks were observed, when the amplified mRNAs were analyzed with the Bioanalyzer.

The presence of high levels of MHV vRNA affected the microarray analyses, depending on the microarray platform used. On the one hand, the high amounts of vRNA were shown to result in a large number of false-positive hits probably as a result of mishybridization of vRNAs with specific oligonucleotides of the 70-mer oligonucleotide arrays. Indeed, small stretches (10–15 nt) of homologous sequences were observed between vRNAs and the oligonucleotides corresponding with the false-positive hits. On the other hand, the same high number of false positive hits was not observed with the Affymetrix arrays, which contain multiple, much shorter, oligonucleotides per gene. Long nucleotide probes, rather than short (25-mers and shorter), demonstrate less non-specific binding, as is often observed when partially degraded RNA samples are used, due to better hybridization and wash stringency [20]. More importantly, however, Affymetrix GeneChips^® ^expression values are calculated by analyzing at least 11 different probes that anneal to the 3' end of each target transcript. Thus, cross-hybridization of vRNA to only one of these probes may not result in an over-estimation of induced gene expression, and will be considered an outlier. However, also for the Affymetrix arrays, the capture and removal of the 'contaminating' vRNAs clearly increased the sensitivity and accuracy of the microarray experiments. Removal of vRNA lowered the threshold for the detection of differentially transcribed genes, thereby identifying potentially important genes for the understanding of virus-host interactions.

MHV-A59 vRNAs were removed from the total RNA samples using the GlobinClear kit (Ambion) with the use of an alternative oligo capture mix, containing three 5' biotinylated oligo's that are complementary to either the 5' leader sequence, the N gene, or the 3' UTR of the MHV-A59 genome. With each of the three oligo's we could target all vRNAs produced in an MHV-infected cell (Fig. 1), resulting in a 90% reduction of vRNA present in the total RNA sample. Care was taken in the design of the oligos in order to minimize removal of cellular RNAs. However, the procedure may be optimized further by using alternative capture oligos. This method proved to be better than a Rnase H digestion protocol, in which we tried to remove the poly(A) tail from the vRNAs specifically by using the MHV-specific oligo targeting the 3'UTR. RNAse H would digest vRNA bound to the DNA oligo, thereby preventing subsequent vRNA amplification during cRNA synthesis. Although we could indeed see some improvement of the microarray performance after RNase H digestion (data not shown), the improvement was less pronounced compared to direct vRNA removal using the GlobinClear system.

The GlobinClear kit has been designed to remove globin mRNAs from total blood RNA samples. Expression array data generated from whole blood total RNA samples are commonly known to have reduced detection sensitivity compared to data from fractionated blood samples [21]. This is mainly caused by the fact that globin mRNA constitutes a large fraction (up to 70%) of the total RNA pool, since globin mRNA is highly expressed in red blood cells and reticulocytes. Microarray analysis has shown that the high amounts of globin mRNA transcripts resulted in decreased sensitivity and increased variation. Similarly, depletion of these abundant 'contaminating' transcripts from the whole blood total RNA samples also resulted in increased sensitivity [22-25]. This observed increase in detection sensitivity after targeted RNA depletion could well be a result of decreased competition between the abundant RNA and cellular mRNA for access to the amplification reagents. Removal of these "contaminating" RNAs will than lead to an increased labeling of host mRNAs, which is detectable by microarray hybridizations. In our experiments, the quality assessment metrics of the affymetrix arrays indeed showed an increase in the number of detected probe sets after vRNA depletion (see Additional file 2).

High levels of 'contaminating' vRNAs, are not only expected in RNA samples derived from coronavirus-infected cells, but also from cells infected with other RNA viruses. For flaviviruses, the vRNAs of which do not contain polyadenylated 3'UTRs, mRNA amplification with oligo(dT) primers should be sufficient to diminish the effect of vRNAs on microarray performance. However, the mRNAs synthesized by most other RNA viruses contain poly(A) tails. In case of picorna- or alphaviruses, infection results in the rapid production of high amounts of only 1 or 2 species of polyadenlyated vRNAs [5,6]. Therefore, only a limited set of oligo's is likely to be required to remove these transcripts from total RNA samples by means of the oligo-capture procedure. For orthomyxoviruses, which contain segmented RNA genomes from which different polyadenylated vRNAs are produced [26], a set of oligonucleotides will be required to eliminate all vRNA species. It will be of interest to investigate whether microarray expression profiling studies performed with RNA samples derived from cells infected with other viruses also benefit from the removal of vRNAs.

## Conclusion

In this study we show that the presence of abundant viral RNAs interferes with microarray gene expression profiling, affecting both the accuracy and sensitivity of the procedure. Targeted removal of vRNA improved the microarray analyses significantly on different array platforms.

## Methods

### Cells and viruses

LR7 mouse fibroblast cells [27] were maintained in Dulbecco's Modified Eagle's Medium (DMEM) (Cambrex Bio Science) containing 10% (v/v) fetal calf serum (Bodinco B.V.), 100 U/ml Penicillin, and 100 μg/ml Streptomycin, supplemented with Geneticin G418 (250 μg/ml). MHV strain A59 was grown in and titrated on LR7 cells.

### MHV infection and total RNA isolation

LR7 cells were inoculated with MHV-A59 at a multiplicity of infection (MOI) of 10 TCID_50 _(50% tissue culture infectious doses) per cell, in phosphate buffered saline (PBS) containing 50 μg/ml diethylaminoethyl-dextran (PBS-DEAE). When indicated, the cells were incubated with 20 μg/ml of Actinomycin D (ActD; Sigma-Aldrich) from 1 h prior to infection and maintained in the presence of this drug throughout the experiment. After a 1 h inoculation, the cells were washed and the culture medium was replaced by complete DMEM. One hour later, at 2 h post infection (p.i.), the fusion inhibitory mHR2 peptide (1 μM) was added to the culture medium to inhibit cell-to-cell fusion [28]. Total RNA was isolated from mock or MHV-A59 infected cells at the indicated time p.i. using the TRIzol reagent (Invitrogen). RNA was further purified using the RNeasy mini-kit with subsequent DNaseI treatment on the column (Qiagen). RNA concentration and integrity were determined by spectrometry and by a microfluidics-based platform using a UV-mini1240 device (Shimadzu) and a 2100 Bioanalyzer (Agilent Technologies), respectively.

### vRNA extraction

MHV-A59 vRNA was removed from the total RNA samples using the GlobinClear kit (Ambion Inc.) with the use of an alternative oligo capture mix that contained three 5' biotinylated oligo's, complementary to the 5' leader sequence, the nucleocapsid (N) gene and the 3'untranslated region (UTR) of the MHV-A59 genome. All subsequent steps were performed according to the manufacturer's protocol. After purification, RNA integrity was analyzed as described above. The sequences of the vRNA capture oligo's are listed in Table 1, and the locations of the complementary sequences in the MHV-A59 genome are depicted in Fig. 1.

**Table 1:** vRNA capture mix.

#	Oligonucleotide sequences (5'to 3')	Locations of complementary nucleotides within the MHV-A59 genome
A	Biotin-CTACAAGAGTTTTAGAGTTGAGAGGGTACG	24–53; 5'Leader
B	Biotin-GCACTACGCCATCATCAAGGATCTGAG	30975–31002; N gene
C	Biotin-GGACCTTGCTAACTTCTCTCACACATTCTC	31187–31216; 3'UTR

### cRNA synthesis, labeling, and hybridization onto microarrays

#### 70-mer oligonucleotide arrays

mRNA was amplified from 1 μg of total RNA by cDNA synthesis with oligo(dT) double-anchored primers, followed by *in vitro *transcription using Amino Allyl MessageAmp™ II kit (Ambion) as described previously [29]. During transcription, 5-(3-aminoallyl)-UTP was incorporated into the single stranded cRNA. Cy3 and Cy5 NHS-esters (Amersham Biosciences) were coupled to 2 μg cRNA. RNA quality was monitored after each successive step using the methods described above. A Mouse Array-Ready Oligo set (version 3.0) was purchased (Operon) and printed on Corning UltraGAPS slides. Mouse slides containing 35,000 spots (32,101 70-mer oligonucleotides, and 2,891 control spots) were hybridized with 1 μg of each alternatively labeled cRNA target at 42°C for 16–20 h using LifterSlips (Erie Scientific) and Corning Hybridization Chambers [30]. After hybridization the slides were washed extensively and scanned using the Agilent G2565AA DNA Microarray Scanner.

#### Affymetrix arrays

mRNA amplification was performed using the MessageAmp™ II Biotin *Enhanced *kit from Ambion, according to the manufacturer's instructions. Briefly, first-strand cDNA was synthesized by a reverse transcription reaction with T7 oligo(dT) primers. Second-strand cDNA was synthesized with the DNA polymerase mix supplied by the kit to provide double-stranded DNA template for *in vitro *transcription. Biotinylated amplified cRNA was produced by T7 RNA polymerase, after which the cRNA targets were hybridized to Mouse Genome 430 2.0 arrays (Affymetrix, Santa Clara, USA). After extensive washing and subsequent staining, the arrays were scanned using an Affymetrix Genechip^® ^Scanner 3000. All steps were performed as recommended by the manufacturer.

### Microarray data analysis

#### 70-mer oligonucleotide arrays

Images were quantified and background corrected using Imagene 5.6 software. The data were normalized using Lowess print-tip normalization as described previously [19]. To identify the genes that were significantly different within each experiment, a one-class Significance Analysis of Microrarrays (SAM) [31] was performed on the average of independent dye-swap hybridizations (n = 6), using a false discovery rate (FDR) of 1%. To increase the confidence level, a cut-off at a 2-fold change in expression was applied. The data were subjected to Genespring 7.2 software (Agilent Technologies) for further analysis.

#### Affymetrix arrays

Images were quantified with Affymetrix Microarray Suite 5.0 (MAS 5.0) software. Quality control (QC) metrics for all arrays are provided as an additional file (see Additional file 2). The robust multichip average (RMA) method [32] was used to process all arrays as a single experiment. A 2-Way ANOVA was used to identify genes that were differentially expressed under the different experimental conditions. The FDR was controlled at 5% using the Benjamini-Hochberg (BH) step-up procedure [33]. To increase the confidence level, a cut-off at a 2-fold change in expression was applied. Hierarchical clustering was performed using average linkage clustering with Euclidean Distance. All analyses were performed using Partek^® ^GS software (Copyright, Partek Inc.).

### ArrayExpress accession numbers

MIAME-compliant data in MAGE-ML format has been submitted to the public microarray database ArrayExpress [34]. Note that the array data obtained from both platforms are submitted as a single experiment. Accession numbers: array designs, A-UMCU-7 and A-AFFY-45; and gene expression data of MHV-infected LR7 cells, E-MEXP-1373. Also included are complete descriptions of protocols for total RNA isolation and mRNA amplification, P-MEXP-34397; vRNA depletion, P-MEXP-114798; cRNA labeling, P-MEXP-34400, P-MEXP-35534, P-MEXP-8712; array hybridization and washing of slides, P-MEXP-34401, P-AFFY-6; scanning of slides, P-MEXP-34430; and data normalization, P-MEXP-34431, P-MEXP-120375.

### Quantitative RT-PCR

Altered mRNA expression levels of several genes, which were identified by microarray analysis, were verified by quantitative reverse transcription (RT)-PCR using TaqMan^® ^Gene Expression assays (Applied Biosystems), according to the manufacturer's instructions. Note that from the different groups shown in Additional file 1 (i.e. false positive hits, additional hits, and hits by both platforms and both methods) genes were randomly selected for RT-PCR validation. Reactions were performed using an ABI Prism 7000 sequence detection system. The comparative Ct-method was used to determine the fold change for each individual gene. The housekeeping gene GAPDH was used as a reference in all experiments. The amounts of viral genomic and subgenomic RNA were determined by quantitative RT-PCR as described previously [35,36].

## Authors' contributions

MR, PW, and DB conducted all the experiments. MR wrote the manuscript. RAS, PJMR, and CAMdeH coordinated the research efforts and assisted with writing the manuscript. All authors read and approved the final manuscript.

## Supplementary Material

Additional file 1 **Validation of microarray data generated by different platforms**. Differential expression of a selection of genes after infection with MHV at 6 h p.i. is shown as determined by two different microarray platforms, with or without vRNA depletion, and by quantitative RT-PCR. Note that the genes from the different groups (i.e. false positive hits, additional hits, and hits by both platforms and both methods), were randomly selected for RT-PCR validation. Click here for file

Additional file 2 **QC metrics of Affymetrix Genechips^®^**. Reported QC metrics are shown for each individual array (Chip ID), including scale factor (SF), noise (RawQ), average background signal (Bg Avg), average noise signal (Noise Avg), number of probe sets detected (#P), percent probe sets detected (%P), mean signal for all probe sets (Signal(All)), and β-actin and GAPDH 5'/3' ratios. Click here for file
