# Supplementary material for: Improved microarray gene expression profiling of virus-infected cells after removal of viral RNA
Source: BMC Genomics. 2008 May 14;9:221. doi: 10.1186/1471-2164-9-221 (PMC2397413; doi:10.1186/1471-2164-9-221)
Supplement: Additional file 1 — Validation of microarray data generated by different platforms. Differential expression of a selection of genes after infection with MHV at 6 h p.i. is shown as determined by two different microarray platforms, with or without vRNA depletion, and by quantitative RT-PCR. Note that the genes from the different groups (i.e. false positive hits, additional hits, and hits by both platforms and both methods), were randomly selected for RT-PCR validation. [file 1471-2164-9-221-S1.pdf]

Supplemental Table S1.

		Fold induction				
**False-positive hits:**		Standard	vRNA depletion	Standard	vRNA depletion	RT-PCR
Gene ID	Name	70-mer arrays		Affymetrix arrays		n.d*/n.t**
NM_016808	Usp2	**310.3**	**70.1**	<2	<2	**1.2**
NM_001001809	Olfr218	**210.0**	**12.1**	<2	<2	n.d.
NM_010915	Klk1b4	**209.2**	**22.2**	<2	<2	n.d.
NM_012013	Figla	**162.7**	**23.1**	<2	<2	n.d.
NM_130886	Card14	**9.8**	<2	<2	<2	**1.6**
NM_029562	Cyp2d26	**9.9**	<2	<2	<2	n.d.
NM_175305	Lrrc19	**9.9**	<2	<2	<2	n.d.
NM_174854	Disc1	**9.7**	<2	<2	<2	n.d.
NM_007954	Es1	**9.5**	<2	<2	<2	n.d.
NM_010333	Edg5	**4.8**	<2	**5.5**	<2	**1.0**
NM_178403	Pus7	<2	<2	**4.4**	<2	**1.2**
NM_181400	Wdr47	<2	<2	**2.3**	<2	**1.5**
NM_133753	Errfi1	<2	<2	**2.1**	<2	**1.5**

*n.d. = not detectable, **n.t. = not tested

Supplemental Table S1. continued

		Fold induction				
**Additional hits:**		Standard	vRNA depletion	Standard	vRNA depletion	RT-PCR
Gene ID	Name	70-mer arrays		Affymetrix arrays		n.d*/n.t**
NM_010234	Fos	<2	**2.1**	**14.5**	**16.5**	**27.5**
NM_008390	Irf1	<2	**2.4**	**2.2**	**2.4**	**2.8**
NM_008562	Mcl1	<2	**2.3**	**2.1**	**2.0**	**2.1**
NM_013642	Dusp1	<2	**2.4**	**2.6**	**3.8**	n.t.
NM_009344	Phlda1	<2	**2.5**	**2.0**	**2.6**	n.t.
NM_178392	Snapc1	<2	**2.1**	**2.0**	**2.4**	n.t.
NM_010495	Id1	<2	**2.1**	**2.5**	**2.6**	n.t.
NM_008235	Hes1	<2	**2.0**	**2.7**	**2.7**	n.t.
NM_029092	Rg9mtd1	<2	**2.0**	**2.0**	**2.1**	n.t.
NM_010496	Id2	<2	**2.0**	<2	**2.7**	**2.8**
NM_172154	Lcor	<2	**2.0**	<2	**2.7**	**4.2**
NM_011498	Bhlhb2	<2	**2.1**	<2	**2.5**	n.t.
NM_010591	Jun	<2	**2.0**	<2	**2.3**	n.t.
NM_008871	Serpine1	<2	**2.3**	<2	<2	**2.2**
NM_010804	Mllt10	<2	<2	<2	**2.6**	**2.1**

*n.d. = not detectable, **n.t. = not tested

Supplemental Table S1. continued

		Fold induction				
**Hits by both platforms and both methods:**		Standard	vRNA depletion	Standard	vRNA depletion	RT-PCR
Gene ID	Name	70-mer arrays		Affymetrix arrays		n.d*/n.t**
NM_010907	Nfkbia	**2.6**	**2.7**	**2.9**	**3.5**	**3.4**
NM_022331	Herpud1	**2.4**	**2.6**	**4.3**	**5.7**	**5.1**
NM_008176	Cxcl1	**5.9**	**7.3**	**6.0**	**6.1**	**10.8**
NM_009140	Cxcl2	**3.8**	**6.3**	**6.3**	**7.7**	**7.2**
NM_013692	Klf10	**4.7**	**6.1**	**5.4**	**5.8**	n.t.
NM_013602	Mt1	**2.2**	**3.1**	**3.5**	**4.3**	n.t.
NM_011803	Klf6	**2.6**	**2.7**	**2.6**	**2.9**	n.t.
NM_153159	Zc3h12a	**2.1**	**3.4**	**2.4**	**2.8**	n.t.
NM_015786	Hist1h1c	**2.6**	**4.4**	**5.2**	**7.5**	n.t.
NM_133662	Ier3	**3.2**	**3.4**	**4.7**	**4.9**	n.t.
NM_008321	Id3	**2.6**	**2.8**	**2.9**	**3.2**	n.t.

*n.d. = not detectable, **n.t. = not tested
