# Supplementary material for: Improved microarray gene expression profiling of virus-infected cells after removal of viral RNA
Source: BMC Genomics. 2008 May 14;9:221. doi: 10.1186/1471-2164-9-221 (PMC2397413; doi:10.1186/1471-2164-9-221)
Supplement: Additional file 2 — QC metrics of Affymetrix Genechips®. Reported QC metrics are shown for each individual array (Chip ID), including scale factor (SF), noise (RawQ), average background signal (Bg Avg), average noise signal (Noise Avg), number of probe sets detected (#P), percent probe sets detected (%P), mean signal for all probe sets (Signal(All)), and β-actin and GAPDH 5'/3' ratios. [file 1471-2164-9-221-S2.pdf]

Supplemental Table S2.

(Chip ID)	SF	RawQ	Bg Avg	Noise Avg	**#P**	**%P**	Signal (All)	Gapdh Ratio	β-Actin Ratio
**MHV #A**	7,56	1,10	34,31	1,31	**19191**	**42,55**	791,98	0,62	1,82
**MHV #B**	7,10	1,10	33,45	1,30	**19772**	**43,84**	790,72	0,59	1,65
**MHV #C**	9,97	0,88	28,83	1,04	**18512**	**41,05**	800,34	0,65	1,59
mock #A	5,25	0,92	29,65	1,14	20432	45,30	801,53	0,67	1,11
mock #B	3,50	1,10	33,61	1,47	21362	47,36	779,64	0,68	1,08
mock #C	3,66	1,11	34,76	1,44	21311	47,25	774,95	0,69	1,14
**MHV (vRNA depleted) #A**	4,68	1,00	32,44	1,20	**21265***	**47,15***	734,54	0,85	1,81
**MHV (vRNA depleted) #B**	5,28	0,96	30,42	1,21	**20888***	**46,31***	750,52	0,72	1,59
**MHV (vRNA depleted) #C**	6,44	1,01	32,21	1,21	**20168***	**44,72***	757,22	0,85	1,92
mock (vRNA depleted) #A	4,77	0,96	31,21	1,20	20722	45,95	797,83	0,79	1,26
mock (vRNA depleted) #B	4,99	1,05	34,41	1,26	20611	45,70	789,74	0,77	1,29
mock (vRNA depleted) #C	3,73	1,06	33,42	1,36	21239	47,09	779,72	0,73	1,19

*Note that the number/percentage of detected probe sets (#P and %P) is increased in the arrays hybridized with cRNA from MHV-infected cells after vRNA depletion. The other QC parameters indicate that the generated data are of high quality.
